# First detection of Jingmen tick virus in Corsica, France and development of a real time detection system for multiple tick-associated jingmenviruses

**DOI:** 10.21203/rs.3.rs-4136487/v1

**Published:** 2024-03-27

**Authors:** Vincent Cicculli, Agathe M. G. Colmant, Geraldine Piorkowski, Rayane Amaral, Apolline Maitre, Dorine Decarreaux, Laurence Thirion, Gregory Moureau, Alessandra Falchi, Xavier de Lamballerie, Remi N. Charrel, Nazli Ayhan

**Affiliations:** Unite des Virus Emergents (UVE: Aix-Marseille Univ, Universita di Corsica, IRD 190, Inserm 1207, IRBA); Unite des Virus Emergents (UVE: Aix-Marseille Univ, Universita di Corsica, IRD 190, Inserm 1207, IRBA); Unite des Virus Emergents (UVE: Aix-Marseille Univ, Universita di Corsica, IRD 190, Inserm 1207, IRBA); Unite des Virus Emergents (UVE: Aix-Marseille Univ, Universita di Corsica, IRD 190, Inserm 1207, IRBA); ANSES, INRAE, Ecole Nationale Vétérinaire d’Alfort, UMR BIPAR, Laboratoire de Santé Animale; Unite des Virus Emergents (UVE: Aix-Marseille Univ, Universita di Corsica, IRD 190, Inserm 1207, IRBA); Unite des Virus Emergents (UVE: Aix-Marseille Univ, Universita di Corsica, IRD 190, Inserm 1207, IRBA); Unite des Virus Emergents (UVE: Aix-Marseille Univ, Universita di Corsica, IRD 190, Inserm 1207, IRBA); Unite des Virus Emergents (UVE: Aix-Marseille Univ, Universita di Corsica, IRD 190, Inserm 1207, IRBA); Unite des Virus Emergents (UVE: Aix-Marseille Univ, Universita di Corsica, IRD 190, Inserm 1207, IRBA); Unite des Virus Emergents (UVE: Aix-Marseille Univ, Universita di Corsica, IRD 190, Inserm 1207, IRBA); Unite des Virus Emergents (UVE: Aix-Marseille Univ, Universita di Corsica, IRD 190, Inserm 1207, IRBA)

## Abstract

Jingmen tick virus (JMTV) is a recently discovered segmented RNA virus, closely related to flaviviruses. It was identified for the first time in 2014, in China and subsequently in Brazil. Following this discovery, JMTV-related sequences have been identified in arthropods, vertebrates (including humans), plants, fungus and environmental samples from Asia, America, Africa, Europe and Oceania. Several studies suggest an association between these segmented flavi-like viruses, termed jingmenviruses, and febrile illness in humans. The development of rapid diagnostic assays for these viruses is therefore crucial to be prepared for a potential epidemic, for the early detection of these viruses via vector surveillance or hospital diagnosis. In this study, we designed a RT-qPCR assay to detect tick-associated jingmenviruses, validated it and tested its range and limit of detection with six tick-associated jingmenviruses using *in vitro* transcripts. Then we screened ticks collected in Corsica (France) from different livestock species, in order to determine the distribution of these viruses on the island. In total, 6,269 ticks from eight species were collected from 763 cattle, 538 horses, 106 sheep and 218 wild boars and grouped in 1,715 pools. We report the first detection of JMTV in Corsica, in *Rhipicephalus bursa, Hyalomma marginatum* and *R. sanguineus* ticks collected from cattle and sheep. The highest prevalence was found in the *Rhipicephalus* genus. The complete genome of a Corsican JMTV was obtained from a pool of *Rhipicephalus bursa* ticks and shares between 94.7% and 95.1% nucleotide identity with a JMTV sequence corresponding to a human patient in Kosovo and groups phylogenetically with European JMTV strains. These results show that a Mediterranean island such as Corsica could act as a sentinel zone for future epidemics.

## Introduction

Jingmen tick virus (JMTV) is a segmented RNA virus which was identified for the first time in 2014 in ticks from the Jingmen region of the Hubei province in China and simultaneously from the Mogiana region of Brazil^1,2^. The genome comprises four segments of positive-sense single-stranded RNA. Segments 1 and 3 encode non-structural proteins, genetically and functionally close to the non-structural proteins NS3 and NS5 of members of the *Flavivirus* genus in the *Flaviviridae* family. Segments 2 and 4 encode putative structural proteins, which are not as closely related to flavivirus structural proteins as the non-structural proteins seem to be^2^.

Following this discovery, JMTV RNA was detected in arthropods (including ticks within the *Rhipicephalus, Haemaphysalis, Ixodes, Dermacentor, Amblyomma, Hyalomma* genera), reptiles and mammals (including cattle and humans) from all continents, alongside other related segmented flavi-like virus sequences termed jingmenviruses^1–7^. The jingmenvirus sequences group phylogenetically into two clades: tick-associated jingmenviruses (also found in vertebrates), and sequences detected from insects (type species: Guaico Culex virus, from Culex mosquitoes), crustaceans, plants and fungi^8^.

Two tick-associated jingmenviruses have been found in Europe: JMTV and Alongshan virus (ALSV). JMTV has been detected in humans from Kosovo, in field-collected ticks from Türkiye and Romania, and in an *Aedes albopictus* mosquito laboratory colony in Italy, while ALSV was found in ticks from Finland, France, Germany and Switzerland^3,9–16^. Concurrently, these are the only two jingmenviruses which have been found in humans: JMTV and ALSV-derived RNA and antibodies were detected patients with tick bites and febrile illness in China and Kosovo^5,9,17^.

The tick-associated jingmenvirus clade also includes Yanggou tick virus (YGTV) found in ticks from China and Russia, Takachi virus (TAKV) in ticks from Japan, Pteropus lylei jingmenvirus (PLJV) in bats from Cambodia, a partial genome from mice from the United States of America (Peromyscus leucopus jingmenvirus) and two divergent full genome sequences from cattle feces and soil samples from China (Guangdong jingmen-like virus, GJLV and Hainan jingmen-like virus) ^3,18–22^.

To our knowledge, no analytically validated molecular assay is available for tick-associated jingmenviruses. Such an assay would allow to shed some light on the vector competence of different tick species and help formally elucidate the transmission cycle of jingmenviruses, which would in term facilitate the prediction of virus introduction into new territories. The aim of this study was to design and validate a real-time RT-PCR assay for the detection of tick-associated jingmenviruses, and to generate epidemiological data in Corsica, a French Merditerranean island, by screening ticks collected from farmed and wild animals on the island. We chose Corsica because: i) many genera of ticks, such as *Ixodes, Hyalomma, Dermacentor, Haemaphysalis, Rhipicephalus* and *Amblyomma*, are present^23–25^ and ii) the circulation of zoonotic diseases in Corsica is facilitated by the widespread practice of mixed livestock farming, the presence of avian migration corridors, and strong interactions between livestock, wildlife and human populations^23,26^.

## Material and Methods

### Molecular detection system design

We aligned all sequences of JMTV, ALSV, YGTV available at the time of design (14th of June 2021) from the taxonomy browser of the National Center of Biotechnology Information (NCBI) using the L-INS-I algorithm implemented in MAFFT version 7. Two real-time RT-qPCR assays were designed targeting conserved regions of segments 1 and 2 based on the generated alignment (Table 1). In particular, segment 2 was selected due to its lack of homology with other virus sequences, in an effort to prevent non-specific amplification.

### Molecular detection system selection

To ensure that the RT-qPCR detection systems targeting segments 1 and 2 were functional, a 0.1 μg/μL DNA plasmid synthetized by Geneart (ThermoFisher) containing the regions targeted by the systems in JMTV was serially diluted 10-fold from 100 pg/μL to 0.1 pg/μL and used as template in a qPCR assay (Superscript III, ThermoFisher).

#### Generation of *in vitro* transcribed RNAs

*In vitro* transcripts (IVT) were generated as positive controls and to evaluate the sensitivity and range of detection of the RT-qPCR systems. Plasmids were synthetized by Geneart containing the region of segment 2 amplified by the gTJ-seg2 system, for six jingmenviruses: JMTV (MH133315), ALSV (MN095520), YGTV (MH688530), TAKV (LC628181), PLJV (MN095532) and GJLV (MW896894) (sequences available in Supplementary Table S1). An exogenic NotI hybridization sequence was incorporated to detect potential laboratory contamination, and an actin sequence was also included as another target to quantify the IVT (see Supplementary Table S1). The plasmids were *in vitro* transcribed into RNA using Megashortscript^™^ T7 transcription kit (Invitrogen-Thermo Fisher) with Turbo DNase to remove DNA. RNA transcripts were purified using MEGAclear^™^ Purification of transcription reaction kit (Invitrogen-Thermo Fisher Scientific). A Thermo Scientific^™^ NanoDrop^™^ was used to determine the RNA concentration in ng/μL, converted to RNA copies/μL using the molecular weight of the molecule, calculated with the AAT Bioquest calculator online.

### Determining the range and limit of detection of the gTJ-seg2 system

The six IVTs were serially diluted 1:3 with dilutions ranging from 10^7^ RNA copies/μL to 15 RNA copies/μL, and used as templates in RT-qPCR using gTJ-seg2, with 4 to 8 replicates. All IVTs were tested with the actin system to confirm their reactivity. The cycle threshold (Ct) values obtained were graphed relative to the IVT concentration in logarithmic scale. A Ct value > 40 was considered negative. IVTs negative for all dilutions under 10^7^ RNA copies/μL were tested further, with dilutions starting from 10^10^ RNA copies/μL.

SPSS Statistics software version 24 (IBM) was used to determine the lower limit of detection (LOD), defined as the lowest concentration of RNA achieving a 95% hit rate (LOD95). GraphPad Prism 9.4.1 was used to estimate a linear regression and 95% confidence intervals between the Ct values < 40 obtained with gTJ-seg2 and the RNA copies / μL.

### Real time RT-qPCR

Real time RT-qPCR was performed using QuantiFast Probe RT-PCR kit (Qiagen), on a QuantStudio^™^ 3 real-time PCR system, software version “V1.5.1”. Primers and probes were synthetized by Sigma-Aldrich, Merck KGaA. Reactions were set up with 12.5 μL buffer, 0.25 μL RT enzyme, 0.5 μL Rox, 800 nM of each primer and 200 nM of probe for a final volume of 25 μL. The optimal cycling conditions were: 50°C for 10 min; 95°C for 5 min; 45 cycles of 95°C for 10 sec and Tm for 30 sec, with Tm = 57°C for segment 1, Tm = 55°C for segment 2 and Tm = 60°C for actin. Probes were labeled with FAM dye and Tamra quencher.

### Sample collection

**Figure 1:**
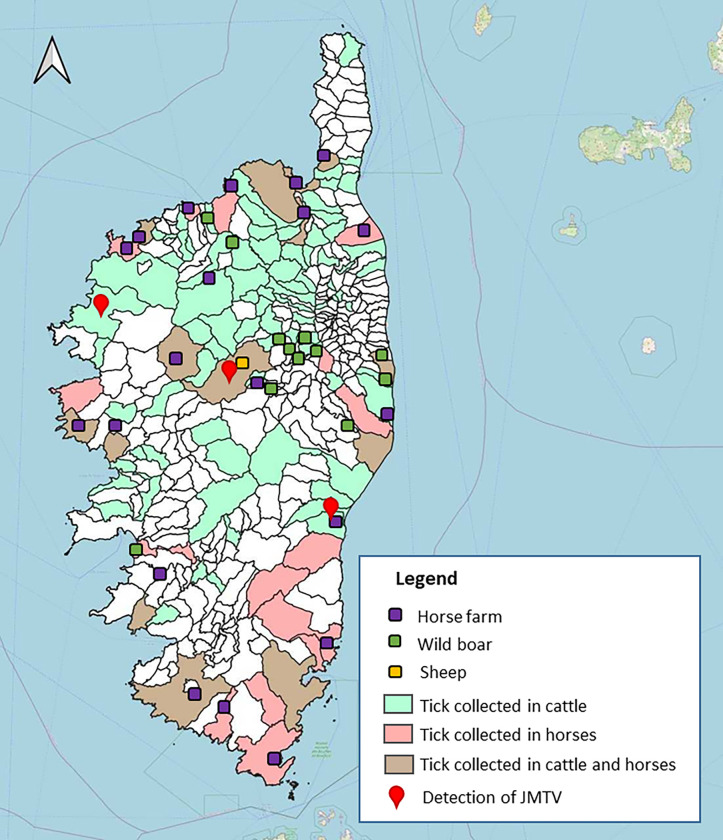
Map of Corsica indicating tick collection sites and positive detection of JMTV RNA in ticks of sites inspected. Colour coded by vertebrate host.

Several types of vertebrate animals were inspected for adult ticks between August 2018 and June 2020, all over Corsica (Fig. 1); 763 bovine cattle from various farms were inspected from January 2019 to June 2020 at the main active slaughterhouse in Corsica, in Ponte-Leccia and; 657 horses were inspected several times between March and August 2019, and in May and June 2020, after each riding excursion in the natural environment across Corsica. Ticks were collected from 218 wild boars in the northeast of Corsica from August to December (hunting season) in 2018 and 2019. A total of 107 sheep were inspected monthly for ticks in May and in June 2020 in a farm located in Corte (Fig. 1).

All ticks were collected and kept alive until morphological identification and storage. Ticks were identified at species level under a stereomicroscope using an identification key, and immediately stored at − 80°C^27^.

### Sample processing and nucleic acid extraction

Ticks collected from the animals were washed once in 70% ethanol and twice in distilled water, then were divided either individually or in monospecific pools of 2 to 10 ticks, according to developmental stage, sex, and animal of collection. They were homogenized in Minimum Essential Medium (MEM) containing 15% of fetal bovine serum, antibiotics (1% penicillin-streptomycin, 1% kanamycin), fungicide (5% amphotericin B) and 1% L-glutamine, using a TissueLyser II (Qiagen, Hilden, Germany) at 30 Hz for 3 min. Nucleic acid extractions were performed on a QIAcube HT (Qiagen) using QIAamp cador Pathogen Mini kits, according to the manufacturer’s instructions. Nucleic acid extracts were eluted in 100 μL buffer and stored at −80°C until they were used as templates in RT-qPCR. The extraction quality was monitored by systematically spiking MS2 bacteriophage and quantifying it by RT-qPCR.

### Complete genome and RT-qPCR amplicon sequencing

One gTJ-seg2-positive pool containing 6 male *R. bursa* ticks (164BOV19) was selected to obtain a complete genome using next generation sequencing (NGS). A total of 200 μL homogenate supernatant was incubated at 37°C for 7 h with 25 U of Benzonase (Novagen) and MgCl_2_. RNA extraction was performed using the Viral RNA mini kit (Qiagen) on the BioRobot EZ1-XL Advanced (Qiagen). Random two-step RT-PCR was performed using tagged random primers in a ProtoScript^®^ II Reverse Transcriptase (New England Biolabs) reaction followed by a Platinum^®^ Taq High Fidelity polymerase (ThermoFisher Scientific) reaction with specific primers^28^. These samples and selected RT-q-PCR amplicons produced as described above using gTJ-seg2 were quantified using the Qubit^®^ dsDNA HS Assay Kit and a Qubit 2.0 fluorometer (ThermoFisher Scientific). All amplicons were sonicated into 200 bp fragments and libraries were built and barcoded using AB Library Builder System (ThermoFisher Scientific). The barcoded fragments were quantified by RT-qPCR using the Ion Library TaqMan^™^ Quantitation Kit (ThermoFisher Scientific) and pooled equimolarly. An emulsion PCR of the pooled fragments was performed and the samples were loaded on an Ion 520 chip (ThermoFisher Scientific) using an automated Ion Chef instrument (ThermoFisher Scientific). Sequencing was performed using the S5 Ion torrent technology (ThermoFisher Scientific) following the manufacturer’s instructions. Reads were trimmed (reads with quality score < 0.99 or length < 100pb were removed, and the first and last 30 nucleotides were removed from all reads) and *de novo* contigs were generated with CLC Genomics Workbench software v.21 (Qiagen). These contigs were blasted (in house BLASTn algorithm) to determine the best reference sequence, and reads were then mapped to that sequence. Parameters for reference-based assembly consisted of match score = 1, mismatch cost = 2, length fraction = 0.5, similarity fraction = 0.8, insertion cost = 3, and deletion cost = 3.

### Phylogenetic analyses

The complete nucleotide sequences of JMTV Corsica 164BOV19 four genome segments obtained in this study were aligned with published tick-associated jingmenvirus sequences using the E-INS-I algorithm implemented in MAFFT version 7^29^. Phylogenetic analyses were inferred for each genomic segment, with maximum likelihood in Mega X, with 100 bootstraps, a general time reversible model gamma distributed^30^.

### Isolation attempts of virus

A total of 10 μL of homogenized tick pool 164BOV19, diluted 1:10 with MEM was injected intracerebrally into 2-day-old OF1 mice (n = 15). This corresponded to a dose of 2.3×10^8 RNA copies per animal. The mice were observed for 14 days for clinical signs of disease, then euthanized by cervical dislocation under general anaesthesia and brains were collected. Nucleic acids were purified from the brain tissues using QIAcube HT with QIAamp 96 Virus QIAcube HT Kit, and tested for presence of JMTV with the segment 2 RT-qPCR described above.

*In vivo* experiments were approved by the French ‘Ministère de l’Enseignement Supérieur, de la Recherche et de l’Innovation’ (APAFIS#9368) and performed in accordance with the French national guidelines and the European legislation covering the use of animals for scientific purposes. All experiments were conducted in a BSL3 laboratory.

## Results

### Molecular detection system selection

In order to ensure that the two systems designed in this study to detect tick-associated jingmenviruses were functional, the plasmid containing the region targeted by both systems was diluted from 100 pg/μL to 0.1 pg/μL and used as template in qPCR assays with gTJ-seg1 or gTJ-seg2. Both systems were indeed functional, gTJ-seg2 was more sensitive than gTJ-seg1 by 3 to 7 Ct (Table 2) and was therefore selected to be used for large-scale screening of all tick pools.

### Range and limit of detection of the gTJ-seg2 system

Tripling dilutions of *in vitro* transcribed RNAs corresponding to increasingly divergent jingmenviruses in the tick-associated clade were used in a RT-qPCR to determine the range of virus sequences detected by gTJ-seg2. A RT-qPCR system detecting actin was used alongside as a positive control for all IVTs.

**Figure 2:**
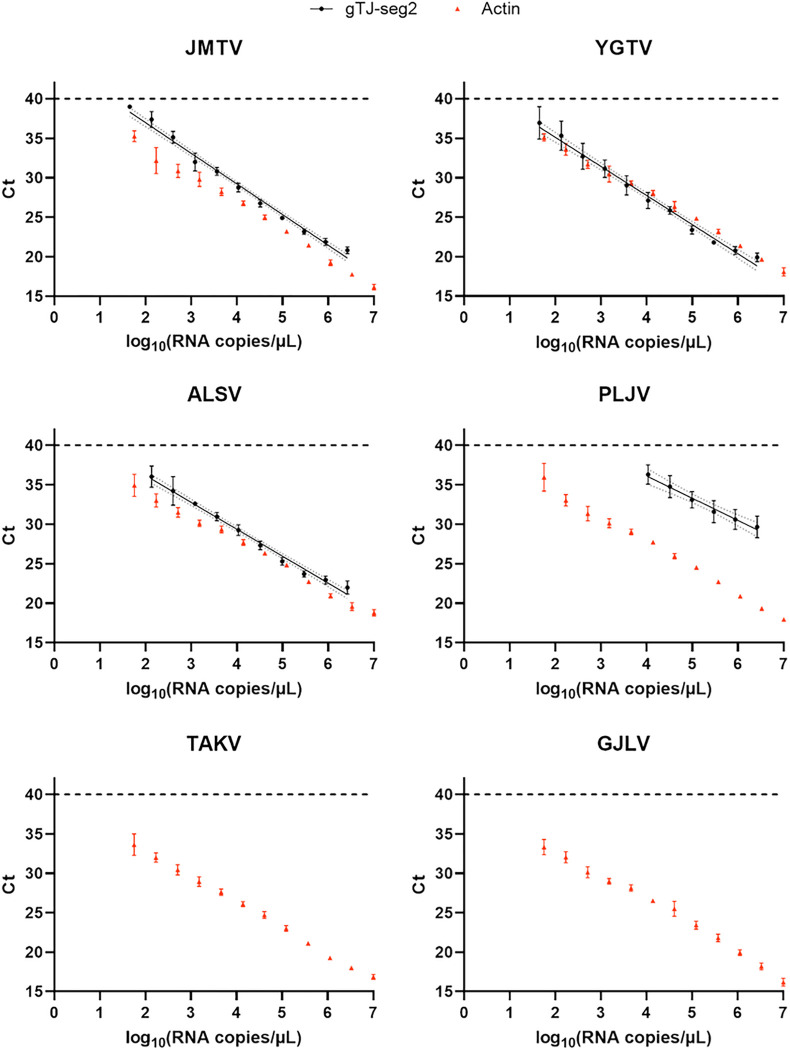
Range and limit of detection of gTJ-seg2. IVTs of six increasingly divergent tick-associated jingmenvirus sequences were serially diluted in multiple replicates and used in RT-qPCR with gTJ-seg2 (black discs, n=4) and an actin system (red triangles, n=8). These graphs represent only the Ct < 40 (cycle threshold) obtained, as a function of the quantity of template used for both systems. All tested dilutions (up to 10^10^ RNA copies /μL) were negative for TAKV and GJLV using gTJ-seg2. Dashed line: limit of detection of the RTqPCR assays: Ct = 40; Straight line: linear regression of gTJ-seg2 data; Dotted lines: 95% confidence intervals, both estimated using GraphPad Prism 9.4.1; error bars represent the standard deviation.

The lowest limits of detection were obtained for YGTV, JMTV and ALSV (140 RNA copies / μL, 245 RNA copies / μL and 298 RNA copies / μL respectively) (Fig. 2, Supplementary Table S2). PLJV was able to be detected at high IVT concentrations (limit of detection: 2.8×10^4^ RNA copies/μL) while TAKV, GJLV remained negative even at 10^10^ IVT RNA copies/μL. All IVTs were detected using the actin system at all tested concentrations.

Ct < 40 were plotted against the RNA copies / μL using GraphPad Prism and a linear regression was estimated (alongside 95% confidence intervals) for the four sequences detected by gTJ-seg2. The linear regression formulas were as follow: JMTV *y* = −3.878 × *x* + 44.72; YGTV *y* = −3.687 × *x* + 42.49; ALSV *y* = −3.410 × *x* + 42.99; PLJV *y* = −2.807 × *x* + 47.33, where *y* is Ct and *x* is log_10_(RNA copies/μL).

**Figure 3:**
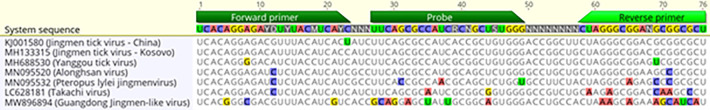
Multiple sequence alignment of the region amplified by gTJ-seg2 in seven reference jingmenvirus sequences. The coloured nucleotides do not match the gTJ-seg2 sequences. Figure generated using Geneious Prime (Version 2023.4).

These results are in line with the *in silico* sequence analysis of these viruses over the region amplified by the segment 2 system (Fig. 3). Indeed, JMTV, YGTV and ALSV have either none or only one mismatch when comparing their sequence with the forward primer, the probe, or the reverse primer. In contrast, PLJV contains three mismatches with the probe and reverse primer, which could explain its higher limit of detection. TAKV presents six mismatches with the reverse primer which could explain why it cannot be amplified by gTJ-seg2. The divergent GJLV cannot be amplified either using gTJ-seg2, as it has three mismatches in the forward primer, eight mismatches over the probe and ten mismatches over the reverse primer.

### Tick Collection and Morphological Identification

In total, 6,269 ticks were collected and 1,715 pools were formed (Table 3). Overall, 3,923 ticks from eight species were collected from 763 cattle, 365 were infested with ticks (45%), and morphologically identified. The most abundant species in cattle was *R. bursa* (n = 2,310; 59.9%), followed by *Hyalomma marginatum* (n = 1,036; 26.4%), *H. scupense* (n = 282; 7.1%), *R. annulatus* (n = 130; 3.3%), *Ixodes ricinus* (n = 75; 1.9%), *R. sanguineus* (n = 74; 1.9%), *Haemaphysalis punctata* (n = 14; 0.4%) and *Dermacentor marginatus* (n = 2). A total of 1,682 ticks from three species were collected from 657 horses, the most abundant species was *H. marginatum* (n = 1,026; 61%), followed by *R. bursa* (n = 629; 37.4%) and *R. sanguineus* (n = 27;1.6%). A total of 626 ticks from three species were collected from 218 wild boards, the main species was *D. marginatus* (n = 613; 97.9%), followed by *H. marginatum* (n = 12; 1.9%) and *R. bursa* (n = 1). A total of 38 *R. bursa* ticks were collected from sheep (Table 3).

The overall adult male to female ratio in the collected ticks was 1.44. In cattle, that ratio was 1.72 (1,421 females, 2,452 males).

### Detection of tick-associated jingmenvirus RNA

In the 1,715 pools tested, tick-associated jingmenvirus RNA was detected in 21 tick pools collected from three cattle and in one tick pool collected from a sheep with a minimum infection rate of 1.22 (MIR).

Out of the 345 cattle infested with ticks, three (n = 345; 1%) had at least one positive tick pool. One bovine had 2/5 pools positive (40%), the second bovine had 13/27 pools positive (48%) and the last bovine had 6/14 pools positive (43%) (Table 4, Supplementary Table S3). These three animals were from three distinct locations and breeding in Corsica (Fig. 1).

Only two of the JMTV positive pools collected from cattle were female ticks while the remaining (n = 19) pools were male, indicating a significantly higher prevalence in male (0.79% [0.49%-1.12%]) than in female ticks (0.14% [0.02%-0.43%]) collected from cattle.

Only one of the 107 sampled sheep had a positive tick pool. That animal had 1/2 (50%) pools positive.

The highest prevalence was found in the *Rhipicephalus* genus with a MIR of 0.58% in *R. bursa* and 0.55% in *R. sanguineus* followed by *H. marginatum* 0.19%. The MIR of positive ticks collected in cattle was 0.54%, more specifically 0.70% for *R. bursa*, 1.16% for *R. sanguineus* and 0.39% for *H. marginatum*.

### Sequence and phylogenetic analyses

We obtained the full genome sequence of JMTV Corsica 164BOV19 from a pool of 6 male *R. bursa* ticks collected from cattle in 2020 (bolded in Table 4) (Genbank accession numbers PP275067-PP275070). The segment sequences shared approximately 95% nucleotide identity (94.0–95.3% depending on the strains and segments) with other European sequences of JMTV (clade II), detected in humans from Kosovo (MH133321-MH133324), and in *R. bursa* from Romania (MW561147-MW561150) and Türkiye (MN486256, MN486261, MN486264, MN486267). JMTV Corsica 164BOV19 was more distantly related (84.4–90.2% depending on the strains and segments) to three other clade II strains, detected in ticks from the French Antilles and Trinidad and Tobago or in a mosquito colony from Italy. JMTV Corsica 164BOV19 is even more distantly related (77.2–82.6% depending on the strains and segments) to sequences from clade I, of Asian, South American and African origins (Table 5). We obtained partial sequences for 15 positive samples, by NGS of RT-PCR amplicons; all were 100% identical at the nucleotide level to the JMTV sequence obtained for 164BOV19.

**Figure 4:**
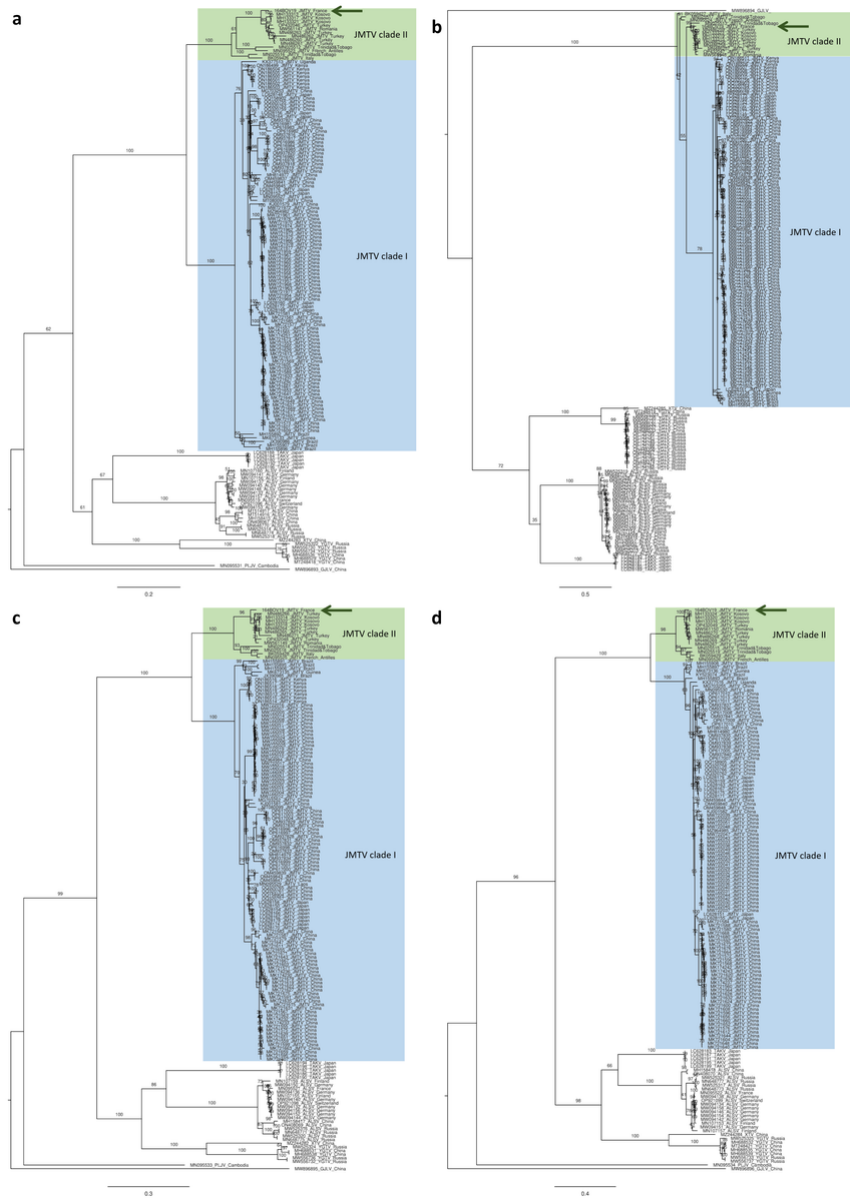
Maximum likelihood analysis of jingmenvirus nucleotide sequences for segments 1 to 4 ORFs (panels a to d respectively). The phylogenetic trees were constructed using the general time reversible model, gamma distributed with 100 bootstraps (branch labels). The scale bar represents the number of nucleotide substitution per site.

The JMTV Corsica 164BOV19 sequences were aligned with published jingmenvirus sequences and phylogenetic trees were built for each segment (Fig. 4). We found that JMTV Corsica 164BOV19 was most closely related to European and Caribbean JMTV sequences (clade II), separately from Asian, South American, and African JMTV strains, which form another clade (clade I).

### Virus isolation

We attempted the isolation of JMTV in new-born mice by inoculating JMTV positive tick homogenate intra-cranially in two to three days old OF1 mice. No clinical signs were observed in the inoculated mice for two weeks. While JMTV RNA was detectable in the inoculum, viral RNA was not detected in the mice brains fourteen days after intra-cranial inoculation using gTJ-seg2 in RT-qPCR, suggesting the lack of virus propagation.

## Discussion

In this study, we designed and characterized two RT-qPCR systems (gTJ-seg1 and gTJ-seg2) targeting segments 1 and 2 of the three tick-associated jingmenviruses published at the time (JMTV, ALSV and YGTV) and we report evidence of circulation of JMTV in ticks collected from cattle in Corsica.

Segment 2 was chosen as a target for the detection and quantification of tick-associated jingmenviruses due to its relatively high homology between target species, the absence of homology with other viruses or living beings^1^ and the lower limit of detection of gTJ-seg2 compared to gTJ-seg1. We showed that gTJ-seg2 can detect several jingmenvirus sequences (JMTV, YGTV, ALSV and PLJV), all in the tick-associated jingmenvirus group. While some RT-qPCR assays have been developed to detect single species of jingmenviruses, to our knowledge, gTJ-seg2 is the first RT-qPCR assay to detect all aforementioned tick-associated jingmenviruses^31^.

Therefore, gTJ-seg2 assay was used to screen ticks, belonging to eight species, collected in Corsica, and successfully detected JMTV in 22 pools of *R. bursa, H. marginatum* and *R. sanguineus*, with the highest JMTV detection rate found in *R. bursa*. JMTV has previously been detected in these tick species from other regions: *R. bursa* from Türkiye and Romania, in *H. marginatum* from Türkiye and in *R. sanguineus* from Türkiye and China^1,10,11,32^. The varying tick numbers and different detection methods prevent direct comparisons with these studies. Taken together, published data to date and our findings seem to indicate an association between JMTV and *Rhipicephalus* ticks, while JMTV can be found in other tick genera, albeit in lower prevalence^10,11^.

The RNA loads detected in Corsican ticks were higher than the limit of detection for JMTV, YGTV and ALSV by a few orders of magnitude (up to 10^7^ RNA copies/μL detected vs a limit of detection around 10^2^ RNA copies/uL) suggesting that YGTV and ALSV would have been detected in those ticks, had they been present. PLJV might have been detected (limit of detection around 10^4^ RNA copies/μL) if present, although this sequence has only ever been detected in bat-derived samples. However, TAKV would not have been detected by our system and would require screening using a virus-specific detection system or a newly developed generic system, based on alignments including this more recently discovered sequence.

We were not successful in isolating JMTV Corsica from new-born mice, despite inoculating each animal with > 10^8^ viral RNA copies. JMTV isolation was previously reported as challenging in both new-born mice and cell culture^1,5^. Elucidating the mechanisms preventing JMTV propagation in laboratory models would allow for a better understanding of viral replication and host-restriction mechanisms and would be crucial in case they emerge as human or veterinary pathogens.

Many questions remain unanswered regarding the natural life cycle of tick-associated jingmenviruses, but there is mounting evidence that they are arthropod-borne viruses. Jingmenviruses have been detected both in male, female, engorged and non-engorged adult ticks as well as non-engorged larvae, which can be evidence for trans-ovarian transmission^18,33^. Our cattle-harvested JMTV-positive ticks corresponded to three animals, and these animals were in contact with up to 13 positive tick pools; this may represent a sign of horizontal transmission from the cattle to the ticks, or of co-feeding transmission from tick to tick, as previously described for tick-borne flaviviruses^3,34,35^. Another argument in favour of the horizontal transmission hypothesis is the presence of jingmenviruses in the salivary glands of ticks and in association with vertebrates, including humans presenting tick bites and febrile illness symptoms, monkeys, rodents, bats, pigs, sheep, cattle and tortoises^5,17^. Moreover, there appears to be a lack of correlation between jingmenvirus sequences and their respective hosts of origin. Instead, phylogenetic clades and geographical distribution are notably associated^18,36^. This pattern is prominently evident in our phylogenetic analyses, incorporating JMTV Corsica within the Caribbean-European JMTV clade (clade II), distinct from the African-Asian-South American clade (clade I). Together, these data align with the hypothesis that jingmenviruses can be transmitted horizontally between ticks and vertebrate hosts.

In light of the strong emerging potential of tick-associated jingmenviruses, the development of a molecular detection tool is important to improve the knowledge on virus circulation by implementing surveillance on vectors and reservoirs, as well as to encourage physicians to consider jingmenvirus infection in patients with undiagnosed febrile illness. Whether the gTJ-seg2 real-time molecular assay, developed in our study, is sensitive enough to be suited for diagnostics in clinical samples from human patients remains to be investigated. The need for serological techniques must also be raised. To the best of our knowledge, we provide here the first evidence of JMTV circulation in Corsica and more widely in the West Mediterranean region, and the first RT-qPCR system validated for the detection of multiple tick-associated jingmenviruses.

## Figures and Tables

**Table 1:** Nucleotide sequences and amplicon size of two generic tick-associated jingmenvirus (gTJ) RT-qPCR systems designed on multiple sequence alignments of segments 1 and 2.

Name	Function	5’ to 3’ sequence	Amplicon size
gTJ-seg1 F	Forward	ATYACNGCYGTYTCYCTNTGGGA	92 bp
gTJ-seg1 R	Reverse	TTGACRT KYTTYAY RTTRGCRTTGAT	
gTJ-seg1 P	Probe	FAM-TGGATGGCCGACCCYGCYATAA-Tamra	
gTJ-seg2 F	Forward	T CACAGGAGAYDTYT ACMT CAYC	76 bp
gTJ-seg2 R	Reverse	AGCGCCGCNTCCGCCCTAG	
gTJ-seg2 P	Probe	FAM-TTCAGCGCCATCRCNGCTSTGGG-Tamra	

**Table 2:** Cycle thresholds (Ct) obtained with gTJ-seg1 and gTJ-seg2 on serial dilutions of plasmids containing both systems’ targets in JMTV.

	gTJ-seg1		gTJ-seg2	
Plasmid concentration	Replicate 1	Replicate 2	Replicate 1	Replicate 2
100 pg/μL	22,9	22,9	17,9	17,6
10 pg/μL	25,8	25,8	22,8	22,8
1 pg/μL	31,7	32,2	24,3	24,5
0,1 pg/μL	35,1	35,3	27,8	27,8

**Table 3:** Ticks collected from farmed and wild animals by tick species and host species.

	Cattle n (%)	Horse n (%)	Boar n (%)	Sheep n (%)	Total n (%)
*R. bursa*	2,310 (59.9)	629 (37.4)	1 (0.2)	38 (100)	2,978 (47.5)
*H marginatum*	1,036 (26.4)	1,026 (61.0)	12 (1.9)	/	2,074 (33.1)
*H scupense*	282 (7.1)	/	/	/	282 (4.5)
*R. annulatus*	130 (3.3)	/	/	/	130 (2.1)
*I ricinus*	75 (1.9)	/	/	/	75 (1.2)
*R. sanguineus*	74 (1.9)	27 (1.6)	/	/	101 (1.6)
*Hae. punctata*	14 (0.4)	/	/	/	14 (0.2)
*D. marginatus*	2 (0.05)	/	613 (97.9)	/	61 5 (9.8)
**Total n (%)**	3,923 (62.6)	1,682 (26.8)	626 (10.0)	38 (0.6)	6,269

R. Rhipicephalus; H. Hyalomma; I. Ixodes; Hae. Haemaphysalis; D. Dermacentor.

**Table 4:** gTJ-seg2-positive tick pool details.

Host type	Host ID	Tick pool ID	Tick species	Ticks/pool	Sex	Location	Date	Ct	RNA copies/μL^†^
**Cattle**	**3339**	**164BOV19** #	***R bursa***	**6**	**M**	**Ventiseli**	**22.05.19**	**16.32**	**3.4×10^7**
		165BOV19*	*R bursa*	6	M			30.68	1.0×10^4
	2005	529BOV20	*R bursa*	6	M	Galeria	26.05.20	18.10	1.2×10^7
		537BOV20*	*R bursa*	6	M			14.76	8.2×10^7
		538BOV20*	*R bursa*	6	M			14.88	7.7×10^7
		539BOV20*	*R bursa*	6	M			19.56	5.5×10^6
		540BOV20*	*R bursa*	6	F			21.55	1.8×10^6
		541BOV20*	*R bursa*	6	M			21.23	2.1×10^6
		542BOV20*	*R bursa*	6	M			20.21	3.8×10^6
		544BOV20*	*R bursa*	6	M			22.36	1.1×10^6
		545BOV20*	*R sanguineus*	1	M			20.96	2.5×10^6
		546BOV20*	*H marginatum*	6	M			22.66	9.6×10^5
		547BOV20*	*H marginatum*	6	M			23.35	6.5×10^5
		550BOV20*	*H marginatum*	5	M			21.45	1.9×10^6
		552BOV20	*H marginatum*	3	F			31.78	5.6×10^3
	2030	580BOV20*	*R bursa*	6	M	Not available	26.05.20	34.51	1.2×10^3
		581BOV20*	*R bursa*	6	M			30.15	1.4×10^4
		582BOV20	*R bursa*	6	M			32.46	3.8×10^3
		583BOV20	*R bursa*	6	M			33.29	2.4×10^3
		584BOV20	*R bursa*	6	M			31.18	7.9×10^3
		590BOV20	*R bursa*	4	M			30.96	8.9×10^3
Sheep	20201	150SHE20*	*R bursa*	2	F	Corte	18.06.20	28.36	3.9×10^4

# Whole genome sequence obtained by random RNA NGS.

* Confirmed to be JMTV by NGS of RT-qPCR amplicon.

† Estimated using the linear regression formula obtained for JMTV (see Fig. 2).

**Table 5:** Percentage nucleotide identity over the open reading frames of the four genomic segments of selected published JMTV strains and JMTV Corsica (164BOV19, Genbank accession numbers PP275067-PP275070).

Location	Host	Strain	Clade	% nt identity (-segment)				Genbank accession numbers
				−1	−2	−3	−4	
Kosovo	Human	2015-A-K15-1A	II	94.8	95.1	94.8	94.6	MH133321-MH133324
Romania	Tick	Tulcea1	II	94.2	95.0	95.3	94.5	MW561147-MW561150
Türkiye	Tick	T14	II	94.0	94.7	94.7	94.5	MN486256, MN486261, MN486264, MN486267
French Antilles	Tick	JMTV/*Rh. microplus/Am. variegatum*/French Antilles	II	86.2	87.4	87.6	87.5	MN095523-MN095526
Trinidad and Tobago	Tick	TTP-Pool-3b	II	85.0	90.2	86.9	86.9	MN025512-MN025515
Italy	Mosquito	RIMINI	II	84.4	87.1	87.1	86.8	BK059426-BK05942
Laos	Tick	JMTV/*Am. testudinarium*/Lao PDR	I	80.2	80.3	79.3	77.5	MN095527-MN095530
Japan	Tick	19EH-IM24	I	80.2	80.2	79.4	77.7	LC628156-LC628159
Brazil	Tick	MGTV/V4/11	I	80.0	79.6	79.2	77.9	JX390985, JX390986, KY523073, KY523074
China	Tick	SY84	I	79.4	81.1	79.2	77.2	KJ001579-KJ001582
Uganda	Monkey	RC27	I	79.3	82.6*	79.1*	77.9	KX377513-KX377516
Kenya	Tick	MT328	I	79.1	80.6	79.6	77.4	ON186499, ON186506, ON186513, ON186520
Guinea	Tick	KITV/2017/1	I	78.3	79.8	79.6	77.7	MK673133-MK673136

* partial sequence

## Data Availability

The sequences of JMTV Corsica 164BOV19 genomic segments were deposited on Genbank under accession numbers PP275067-PP275070.

## References

[1] Qin X.-C. A tick-borne segmented RNA virus contains genome segments derived from unsegmented viral ancestors. Proc Natl Acad Sci U S A 111, 6744–6749 (2014). 24753611 10.1073/pnas.1324194111 PMC4020047

[2] Maruyama S. R. Characterisation of divergent flavivirus NS3 and NS5 protein sequences detected in Rhipicephalus microplus ticks from Brazil. Mem Inst Oswaldo Cruz 109, 38–50 (2014). 24626302 10.1590/0074-0276130166 PMC4005522

[3] Temmam S. Insights into the Host Range, Genetic Diversity, and Geographical Distribution of Jingmenviruses. mSphere 4, e00645–19 (2019). 31694898 10.1128/mSphere.00645-19 PMC6835211

[4] Souza W. M. de Viral diversity of Rhipicephalus microplus parasitizing cattle in southern Brazil. Sci Rep 8, 16315 (2018). 30397237 10.1038/s41598-018-34630-1 PMC6218518

[5] Jia N. Emergence of human infection with Jingmen tick virus in China: A retrospective study. EBioMedicine 43, 317–324 (2019). 31003930 10.1016/j.ebiom.2019.04.004 PMC6557783

[6] Colmant A. M. G., Furlong M. J. & Etebari K. Discovery of a Novel Jingmenvirus in Australian Sugarcane Soldier Fly (Inopus flavus) Larvae. Viruses 14, 1140 (2022). 35746612 10.3390/v14061140 PMC9229714

[7] Ogola E. O. Jingmen Tick Virus in Ticks from Kenya. Viruses 14, 1041 (2022). 35632782 10.3390/v14051041 PMC9147648

[8] Colmant A. M. G., Charrel R. N. & Coutard B. Jingmenviruses: Ubiquitous, understudied, segmented flavi-like viruses. Front Microbiol 13, 997058 (2022). 36299728 10.3389/fmicb.2022.997058 PMC9589506

[9] Emmerich P. Viral metagenomics, genetic and evolutionary characteristics of Crimean-Congo hemorrhagic fever orthonairovirus in humans, Kosovo. Infect Genet Evol 65, 6–11 (2018). 30006045 10.1016/j.meegid.2018.07.010

[10] Bratuleanu B. E. The virome of Rhipicephalus, Dermacentor and Haemaphysalis ticks from Eastern Romania includes novel viruses with potential relevance for public health. Transbound Emerg Dis (2021) doi:10.1111/tbed.14105. 33840161

[11] Dinçer E. Survey and Characterization of Jingmen Tick Virus Variants. Viruses 11, 1071 (2019). 31744216 10.3390/v11111071 PMC6893481

[12] Kuivanen S. Detection of novel tick-borne pathogen, Alongshan virus, in Ixodes ricinus ticks, south-eastern Finland, 2019. Euro Surveill 24, 1900394 (2019). 31290392 10.2807/1560-7917.ES.2019.24.27.1900394 PMC6628756

[13] Stanojević M. Depicting the RNA Virome of Hematophagous Arthropods from Belgrade, Serbia. Viruses 12, E975 (2020). 10.3390/v12090975 PMC7552015 32887342

[14] Ebert C. L. Detection and Characterization of Alongshan Virus in Ticks and Tick Saliva from Lower Saxony, Germany with Serological Evidence for Viral Transmission to Game and Domestic Animals. Microorganisms 11, 543 (2023). 36985117 10.3390/microorganisms11030543 PMC10055853

[15] Stegmüller S., Fraefel C. & Kubacki J. Genome Sequence of Alongshan Virus from Ixodes ricinus Ticks Collected in Switzerland. Microbiol Resour Announc 12, e0128722 (2023). 36779723 10.1128/mra.01287-22 PMC10019301

[16] Stegmüller S., Qi W., Torgerson P. R., Fraefel C. & Kubacki J. Hazard potential of Swiss Ixodes ricinus ticks: Virome composition and presence of selected bacterial and protozoan pathogens. PLoS One 18, e0290942 (2023). 37956168 10.1371/journal.pone.0290942 PMC10642849

[17] Wang Z.-D. A New Segmented Virus Associated with Human Febrile Illness in China. N Engl J Med 380, 2116–2125 (2019). 31141633 10.1056/NEJMoa1805068

[18] Kobayashi D. Detection of Jingmenviruses in Japan with Evidence of Vertical Transmission in Ticks. Viruses 13, 2547 (2021). 34960816 10.3390/v13122547 PMC8709010

[19] Vandegrift K. J. Presence of Segmented Flavivirus Infections in North America. Emerg Infect Dis 26, 1810–1817 (2020). 32687041 10.3201/eid2608.190986 PMC7392405

[20] Chen Y.-M. RNA viromes from terrestrial sites across China expand environmental viral diversity. Nat Microbiol 7, 1312–1323 (2022). 35902778 10.1038/s41564-022-01180-2

[21] Kholodilov I. S. Geographical and Tick-Dependent Distribution of Flavi-Like Alongshan and Yanggou Tick Viruses in Russia. Viruses 13, 458 (2021). 33799742 10.3390/v13030458 PMC7998622

[22] Kholodilov I. S. Distribution and Characterisation of Tick-Borne Flavi-, Flavi-like, and Phenuiviruses in the Chelyabinsk Region of Russia. Viruses 14, 2699 (2022). 36560703 10.3390/v14122699 PMC9780909

[23] Cicculli V. Molecular Detection of Spotted-Fever Group Rickettsiae in Ticks Collected from Domestic and Wild Animals in Corsica, France. Pathogens 8, E138 (2019). 10.3390/pathogens8030138 PMC6789605 31487899

[24] Cicculli V. Molecular screening of Anaplasmataceae in ticks collected from cattle in Corsica, France. Exp Appl Acarol 81, 561–574 (2020). 32728778 10.1007/s10493-020-00527-w

[25] Cicculli V. Molecular investigation of tick-borne pathogens in ixodid ticks infesting domestic animals (cattle and sheep) and small rodents (black rats) of Corsica, France. Ticks Tick Borne Dis 10, 606–613 (2019). 30777731 10.1016/j.ttbdis.2019.02.007

[26] Cicculli V., de Lamballerie X., Charrel R. & Falchi A. First molecular detection of Rickettsia africae in a tropical bont tick, Amblyomma variegatum, collected in Corsica, France. Exp Appl Acarol 77, 207–214 (2019). 30656468 10.1007/s10493-018-00336-2

[27] Walker A. R. Ticks of Domestic Animals in Africa: A Guide to Identification of Species. (Bioscience Reports, 2003).

[28] Stang A., Korn K., Wildner O. & Uberla K. Characterization of virus isolates by particle-associated nucleic acid PCR. J Clin Microbiol 43, 716–720 (2005). 15695669 10.1128/JCM.43.2.716-720.2005 PMC548055

[29] Katoh K. & Standley D. M. MAFFT multiple sequence alignment software version 7: improvements in performance and usability. Mol Biol Evol 30, 772–780 (2013). 23329690 10.1093/molbev/mst010 PMC3603318

[30] Kumar S., Stecher G., Li M., Knyaz C. & Tamura K. MEGA X: Molecular Evolutionary Genetics Analysis across Computing Platforms. Mol Biol Evol 35, 1547–1549 (2018). 29722887 10.1093/molbev/msy096 PMC5967553

[31] Litov A. G., Okhezin E. V., Kholodilov I. S., Polienko A. E. & Karganova G. G. Quantitative Polymerase Chain Reaction System for Alongshan Virus Detection. Methods and Protocols 6, 79 (2023). 37736962 10.3390/mps6050079 PMC10514782

[32] Dinçer E. Several Tick-Borne Pathogenic Viruses in Circulation in Anatolia, Turkey. Vector Borne Zoonotic Dis 22, 148–158 (2022). 35133905 10.1089/vbz.2021.0082

[33] Wu Z. Molecular evidence for potential transovarial transmission of Jingmen tick virus in Haemaphysalis longicornis fed on cattle from Yunnan Province, China. J Med Virol 95, e28357 (2023). 36443647 10.1002/jmv.28357

[34] Jones L. D., Davies C. R., Steele G. M. & Nuttall P. A. A novel mode of arbovirus transmission involving a nonviremic host. Science 237, 775–777 (1987). 3616608 10.1126/science.3616608

[35] Labuda M., Jones L. D., Williams T., Danielova V. & Nuttall P. A. Efficient transmission of tick-borne encephalitis virus between cofeeding ticks. J Med Entomol 30, 295–299 (1993). 8433342 10.1093/jmedent/30.1.295

[36] Guo J.-J. Diversity and circulation of Jingmen tick virus in ticks and mammals. Virus Evol 6, veaa051 (2020). 33976906 10.1093/ve/veaa051 PMC8097133

